# A global perspective of maternal influenza immunization

**DOI:** 10.1016/j.vaccine.2015.08.036

**Published:** 2015-08-28

**Authors:** Philipp Lambach, Joachim Hombach, Justin R. Ortiz

**Affiliations:** Initiative for Vaccine Research, World Health Organization, Geneva, Switzerland

**Keywords:** Maternal, Immunization, Influenza, SAGE, Initiative for Vaccine Research, Policy, Implementation, Safety

The World Health Organization (WHO) influenza vaccine policy recommendations aim to protect vulnerable high-risk groups from severe disease. In a 2012 update of its influenza vaccine position, WHO recommends that countries considering the initiation or expansion of programmes for seasonal influenza vaccination should prioritize pregnant women over other high risk groups (young children, the elderly, persons with certain chronic illnesses, and health care workers) [1]. The recommendation was based on numerous factors, including influenza disease risk in pregnant women and among their young children in the first months after birth, vaccine safety and effectiveness, as well the programmatic opportunities to reach this population in low- and middle-income countries. This review describes the advancements made in the field of maternal influenza immunization since the 2012 WHO policy recommendation, as well as the evidence gaps that remain. We focus on the evidence needs to review maternal influenza immunization in the poorest countries—many of which have not yet introduced influenza vaccine programs.

In 2013, GAVI, the Vaccine Alliance reviewed maternal influenza immunization for possible inclusion in its vaccine investment portfolio for low-income countries. While GAVI opted not to invest in the strategy, its review was generally positive. GAVI vaccine program impact models estimated that maternal influenza immunization could avert around 45 deaths per 100,000 persons vaccinated in GAVI-eligible countries, similar to the expected impact of rubella and cholera immunization strategies [2]. If vaccine exposure decreased preterm births, the estimated impact of the program would be much greater [2]. GAVI noted that the models were based on uncertain mortality and vaccine effectiveness data [2]. Its review also identified other obstacles to a maternal influenza immunization strategy including decreased awareness of severe influenza disease, as well as a need for strengthening policy, regulatory, implementation, and monitoring processes in many low-resource settings to ensure effective vaccine demand, supply, and program sustainability [2].

A comprehensive assessment of the influenza disease burden is available from a systematic review and meta-analysis of influenza-associated acute lower respiratory infections in children <5 years from 43 studies worldwide [3]. The authors estimated that in 2008 there were 28,000–111,500 deaths in children <5 years of influenza-associated acute lower respiratory infections and that 99% of these deaths occurred in developing countries [3]. The study did not determine mortality estimates for the subgroup <6 months, the age that would benefit from maternal immunization. The Institute for Health Metrics and Evaluation (IHME) estimated that globally 1.7% of all deaths under five years of age are caused by influenza (about 15,100 total deaths), although given the limited published data available for children <6 months, the robustness of IHME estimates are unclear [4]. Finally, the Pneumonia Etiology Research for Child Health (PERCH) Project, a multi-country study of children <5 years, has published pilot data suggesting that influenza is not a major contributor to pneumonia [5,6]. The final PERCH study reports are expected soon [7]. More efforts are needed to collect robust data on severe influenza disease in children <6 months, particularly in low-income settings, and to reconcile discordant findings of different studies of influenza disease burden.

There are limited data on the incidence of severe influenza disease among pregnant women globally [8]. A systematic review and meta-analysis of comparative observational studies of pregnant and non-pregnant women with influenza and severe outcomes was recently published [9]. This review found only one study of pregnancy risk associated with seasonal influenza virus infection, while the remaining relevant studies were from the 2009 influenza A (H1N1) pandemic [9]. As the rates of severe influenza outcomes during the 2009 H1N1 pandemic increased in young adults [10], further research is needed to confirm whether severe outcomes in pregnant women are also associated with seasonal influenza virus infection. Contrasting with the findings of observational studies, ecological or modeling studies have demonstrated increased risk of severe seasonal influenza disease in pregnancy [11]. Future research should confirm the risk of severe seasonal influenza disease in pregnancy seen in modeling studies and to reconcile incongruities between the observational and the ecological literature.

Important data supporting WHO recommendations that pregnant women be prioritized for influenza vaccine receipt came from the landmark Mother’s Gift trial in Bangladesh [1,12]. In this trial, pregnant women were immunized with either pneumococcal polysaccharide vaccine (PPV) or seasonal influenza vaccine. They were then followed for respiratory illness with fever, while their newborns were monitored for both respiratory illness with fever as well as laboratory-confirmed influenza. Influenza vaccine receipt was associated with a 36% reduction in respiratory illness with fever among women and a 63% reduction in laboratory-confirmed influenza in their children [12]. This study was an important proof of principle that paediatric respiratory infections can be prevented through maternal immunization strategies in a low-income setting. Limitations included a small sample size, lack of laboratory-confirmed outcomes in the women, use of an active comparator vaccine active against respiratory infections, and influenza vaccine-related analyses were not pre-specified [13]. Encouraged by these results, the Bill & Melinda Gates Foundation (BMGF) invested in three large clinical efficacy trials in Mali, Nepal, and South Africa [14]. The South Africa trial is the first to be published, and it has confirmed the vaccine efficacy findings of Mother’s Gift [15]. Among HIV-uninfected women and their offspring, attack rates for RT-PCR—confirmed influenza in the placebo arm were both 3.6%, and attack rates in the vaccine arm were 1.8% and 1.9%, respectively. Among HIV-uninfected women, influenza vaccine exposure was associated with a 50% decrease in laboratory-confirmed influenza disease in pregnant women and a 49% decrease in laboratory confirmed influenza disease in their children <6 months. [15]. Given the rarity of severe influenza-associated outcomes, it may not be feasible for clinical trials to determine vaccine efficacy against severe influenza disease or to calculate precise baseline rates of severe influenza disease among those exposed to placebo to inform disease burden estimates.

Another intriguing finding from the Mother’s Gift trial was a difference in birth weight between intervention groups, with influenza vaccine-exposed newborns having a significantly higher birth weight than PPV-exposed newborns among the subset of children born during the influenza season [16]. Numerous observational studies have also found associations between vaccine exposure and birth weight, as well as with additional beneficial birth outcomes including decreased small for gestational age (SGA) and preterm births [17]. In GAVI’s impact model, a sensitivity analysis estimated that the number of infant deaths averted by maternal influenza immunization would increase from 170,000 lives to 900,000 lives over a 15 year program in GAVI-eligible countries if vaccine prevention of preterm birth was included in the model [2]. However, the model assumed vaccine exposure prevented 17% deaths from preterm complications [2], an estimate that is likely overly optimistic [18]. In contrast to the Mother’s Gift trial, the South Africa trial found no statistical difference in birth weight, SGA, or preterm birth between vaccine- and placebo-exposed newborns [15].

The three BMGF clinical trials will provide valuable data about vaccine efficacy to prevent influenza disease in three low-resource countries. Given the limitations of clinical trials to determine vaccine efficacy against severe disease outcomes, observational studies have been conducted or are being planned to estimate vaccine effectiveness to prevent influenza hospitalizations [19,20]. These observational data are valuable for policy makers; however, non-clinical trial data should be interpreted with some caution. Twenty years ago, there was tremendous excitement in the influenza vaccine community about observational studies in the elderly that found dramatic benefits of vaccination against severe outcomes [21]. Subsequently, these findings were discredited and attributed to uncontrolled confounding in the observational study designs [22].

Influenza vaccines have been used in pregnant women for decades, and there is a large knowledge base that supports that the vaccines are safe to use in pregnancy. However, pregnant women have been historically excluded from vaccine trials conducted for licensure of new products, as pregnant women are often considered vulnerable due to physiologic changes during pregnancy and are protected by additional regulations [23]. For this reason, there has been few randomized, placebo-controlled vaccine trials conducted with pregnant women. In 2014, the WHO Global Advisory Committee on Vaccine Safety (GACVS) published the outcome of its expert review of inactivated influenza vaccine safety in pregnancy, reporting that it identified no safety signals among pregnant women associated with inactivated influenza vaccine receipt [24]. Recent systematic reviews of vaccine safety studies support the conclusions of GACVS that there is no increased risk of adverse maternal, fetal, or newborn outcomes associated with maternal influenza vaccination [25–27]. Of the three BMGF-sponsored randomized clinical trials of influenza vaccine in pregnant women [14], two include placebo control arms, facilitating vaccine safety evaluations.

From a policy-making perspective, the most important data gap in the field of maternal influenza immunization is the expected impact a vaccine program would have on prevention of mortality and severe disease. To determine anticipated deaths or severe illnesses averted from a maternal influenza immunization strategy, vaccine impact modeling requires severe influenza disease incidence estimates in pregnant women and in children <6 months, as well as estimates of vaccine effectiveness to prevent severe outcomes. The lack of such estimates may limit decisions about vaccine introduction in many countries.

In 2013, SAGE acknowledged delays in development and implementation of maternal influenza immunization policies globally and requested WHO to address obstacles that prevent the development of policies and national implementation efforts [23]. In response, the WHO, jointly with partners, engaged in numerous activities to support evidence-based influenza vaccine policy making and implementation of maternal influenza immunization programs in countries. The WHO Initiative for Vaccine Research (IVR) has a broad portfolio of activities relevant to maternal influenza immunization encompassing three main areas of work: (1) thorough evidence reviews of disease burden and vaccine performance; (2) addressing global barriers to the implementation of maternal immunization; and (3) developing implementation guidance for maternal influenza immunization programs.

IVR is supporting a working group to evaluate influenza data to inform vaccine impact and economic modeling which is reviewing influenza disease risk and morbidity in pregnant women, children <6 months of age, and the fetus, as well as vaccine performance to reduce influenza disease in these groups. The working group has four objectives: (1) to determine key parameters needed for influenza vaccine impact and health economic modeling studies, with a focus on pregnant women and low-resource settings; (2) to determine evidence-based assumptions for these key parameters; (3) to evaluate the quality of existing data; and (4) to provide recommendations to WHO for addressing data gaps. The working group leaders are conducting a series of relevant systematic literature reviews and the findings are presented to the working group to obtain feedback on methodology, analysis, and interpretation. A final report of the working group is anticipated in late 2015.

Barriers to maternal immunization implementation are addressed by IVR through development of technical guidance assisting countries in introducing maternal influenza immunization. WHO is developing health economics guidance for the evaluation of economic burden of influenza disease, influenza vaccine cost effectiveness, and a maternal influenza immunization program costing tool. These tools will enable decision makers in low-resource setting to compare maternal influenza immunization with regards to other health interventions. WHO is developing tools to assess confidence and use of vaccines among pregnant women. Along with the Tailoring Immunization Programmes for influenza (TIP FLU) guide developed by WHO/Europe [28], these tools can help countries to identify reasons for vaccine hesitancy and develop interventions to best address those specific issues. Other implementation issues receiving WHO attention include reviewing information needs for pregnancy/lactation sections of package inserts [29], developing adverse event following immunization guidance for maternal immunization [30], and developing strategies to support year-round availability of influenza vaccine [31].

As a next step, WHO is synthesizing the various tools and guidance for maternal immunization into an implementation guide for best-practices delivery of influenza vaccine to pregnant women. This implementation guide is being produced with the advice of a group of global vaccine delivery experts, and will be adaptable for country-specific needs as well as for future vaccines targeting pregnant women.

In April 2014, SAGE again reviewed maternal influenza immunization. After reviewing ongoing efforts in the field, SAGE encouraged WHO to promote more implementation research to collect generalizable data on the best ways to integrate maternal immunization into routine antenatal care in low-resource settings [32]. Furthermore, SAGE emphasized the importance of the maternal immunization platform, in general, and called upon WHO to affirm its commitment to building the evidence base to strengthen vaccine delivery during pregnancy, as it has great potential for infection prevention in high-risk groups worldwide, even beyond influenza and tetanus. A meeting of global health stakeholders in maternal immunization organized by the Bill and Melinda Gates Foundation in January 2015 further identified the need for a stronger evidence base, including burden of disease, maternal immunization efficacy and safety for mother, fetus and infant, and for implementation research to assess healthcare costs, integrated healthcare approaches in order to evaluate the investment case for manufacturers and donors, policy recommendations, licensure, and vaccine uptake [8]. In March 2015, WHO convened the *Technical Consultation on Maternal Influenza Immunization and Evidence* to identify data gaps in need of further efforts to strengthen uptake of maternal influenza immunization programs. In the consultation, subject matter experts and global stakeholders reviewed influenza disease burden, vaccine performance, vaccine safety, program impact, and implementation issues. In recent years, major advancements have been made in the field, and several critical vaccine clinical trials and reviews of disease burden will be published in 2015 and 2016 [7,33,34]. After review of the relevant evidence, the consensus of participants recommended further data collection on integration of vaccine programs into antenatal care platforms in low-resource settings.

In order to inform widespread implementation of maternal influenza immunization as a strategy, more work needs to be done. In addition to the needed research to better characterize the risk and incidence of severe influenza disease and the effectiveness of vaccine to prevent severe influenza disease, there must be increased focus on ways to optimally deliver vaccines to pregnant women in low-resource settings. Demonstration projects are needed to evaluate different vaccine delivery approaches that integrate into routine care delivery. Delivery methods that are cost effective and do not adversely affect routine immunization programs or antenatal care must be identified. Post-licensure safety surveillance must be implemented. Monitoring and evaluation systems are needed, including strategies to measure the number of severe influenza illnesses reduced by any program. The goal of any implementation research agenda should be to produce sustainable, integrated programs with predictable vaccine supply and manageable delivery, storage, and stock rotations procedures.

Since 2012, there have been many advances in the field of maternal influenza immunization, and more data are expected soon. Many countries, mostly with low or middle income, have not yet introduced this strategy into their national immunization programs. Low-resource countries have multiple competing public health priorities and limited resources. Additional data, particularly regarding the incidence of severe influenza disease in pregnant women and young children and the anticipated impact of maternal influenza immunization, may be necessary to demonstrate the value proposition of maternal influenza immunization in countries considering influenza vaccine policies.
